# Influence of thickening of the inner skull table on intracranial volume measurement in older people

**DOI:** 10.1016/j.mri.2013.01.012

**Published:** 2013-07

**Authors:** N.A. Royle, M.C. Valdés Hernández, S. Muñoz Maniega, B.S. Arabisala, M.E. Bastin, I.J. Deary, J.M. Wardlaw

**Affiliations:** a Brain Research Imaging Centre, Neuroimaging Sciences, University of Edinburgh, Edinburgh, EH4 2XU, UK; b Centre for Cognitive Ageing and Cognitive Epidemiology, University of Edinburgh, Edinburgh, EH8 9JZ, UK; c Scottish Imaging Network, A Platform for Scientific Excellence (SINAPSE) Collaboration, Department of Clinical Neurosciences, The University of Edinburgh, Edinburgh, EH4 2XU, UK; d Department of Psychology, University of Edinburgh, Edinburgh, UK

**Keywords:** Magnetic resonance imaging, Skull, Intracranial volume, Brain atrophy, Ageing

## Abstract

**Introduction:**

It is generally assumed that intracranial volume (ICV) remains constant after peaking in early adulthood. Thus ICV is used as a ‘proxy’ for original brain size when trying to estimate brain atrophy in older people in neuroimaging studies. However, physiological changes in the skull, such as thickening of the frontal inner table, are relatively common in older age and will reduce ICV. The potential influence that inner table skull thickening may have on ICV measurement in old age has yet to be investigated.

**Methods:**

We selected 60 (31 males, 29 females) representative older adults aged 71.1–74.3 years from a community-dwelling ageing cohort, the Lothian Birth Cohort 1936. A semi-automatically derived current ICV measurement obtained from high resolution T1-weighted volume scans was compared to the estimated original ICV by excluding inner skull table thickening using expert manual image processing.

**Results:**

Inner table skull thickening reduced ICV from an estimated original 1480.0 ml to a current 1409.1 ml, a median decrease of 7.3% (Z = − 6.334; p < 0.001), and this reduction was more prominent in women than men (median decrease 114.6 vs. 101.9 ml respectively). This led to potential significant underestimations of brain atrophy in this sample by 5.3% (p < 0.001) and obscured potential gender differences.

**Conclusions:**

The effects of skull thickening are important to consider when conducting research in ageing, as they can obscure gender differences and result in underestimation of brain atrophy. Research into reliable methods of determining the estimated original ICV is required for research into brain ageing.

## 1 Introduction

Head size is strongly influenced by brain growth in childhood and reaches maximum size by early adulthood [1]. It is generally assumed that head size, and therefore intracranial volume (ICV), remains the same from early adulthood to old age. However, age-related skull changes, such as an increase in the thickness of the inner table and overall size of the cranium, have been found [2,3]. Physiological changes of the skull such as hyperostosis frontalis interna (HFI), thickening of the inner table of the frontal region of the skull, have also long been documented in the medical literature [4]. Whereas it is commonly observed by radiologists in older adults, skull thickening is not often mentioned in ageing research, possibly due to the benign nature of the changes [5]. Although the process is benign, some research suggests that, where the increase is very pronounced, dural irritation and pressure atrophy may occur [6]. Case studies of hydrocephalic children [7] and adults with severe brain atrophy [8] suggest that thickening of the inner skull table may occur in response to the reduction in brain volume caused by atrophy or changes in intracranial pressure. A cause of this sporadic thickening is thought to be hormonal as it is most prominently found in post-menopausal women and some studies have found endocrine abnormalities coincidental with HFI [9,4].

In neuroimaging studies, ICV is used as an estimate of peak prior adult brain volume [10–12]. Because it is thought that ICV is not influenced by disease or age-related changes, it is therefore often used to estimate brain atrophy. However, the influence that thickening of the inner skull table may have on measures of ICV, and hence on estimates of brain atrophy and its correlations, have yet to be investigated. In this paper, we investigated the potential influence of inner table skull thickening on measurement of ICV and estimates of brain atrophy in a cohort of community-dwelling older adults.

## 2 Method

### 2.1 Subjects

We randomly selected 60 participants from the Lothian Birth Cohort 1936 [13] who had, on visual inspection, a range of inner skull table thickening from significant to little or no thickening. Study participants (31 males and 29 females) were non-demented, community-dwelling older individuals who underwent cognitive tests and brain MRI between 8th November 2007 and 29th June 2010 at 71.1 to 74.3 years of age (mean 72.7, standard deviation (SD) 0.7 years).

### 2.2 Image acquisition

Structural brain MRI data were obtained from a GE Signa HDxt 1.5 T MRI clinical scanner (GE Healthcare, Milwaukee, WI, USA) using a self-shielding gradient set with maximum gradient strength of 33 m/Tm, and an 8-channel phased-array head coil. The examination included a high resolution T1-weighted (T1W) volume scan, and whole brain T2- (T2W), T2*- (T2*W) and FLAIR-weighted sequences. Full details of the imaging protocol are provided in Wardlaw et al. [14].

### 2.3 Measurement method

All analyses were performed blind to subject details, including gender, on anonymised scans. The scans were aligned to the anterior-posterior commissure (AC-PC) line to improve reproducibility.

**Fig. 1:**
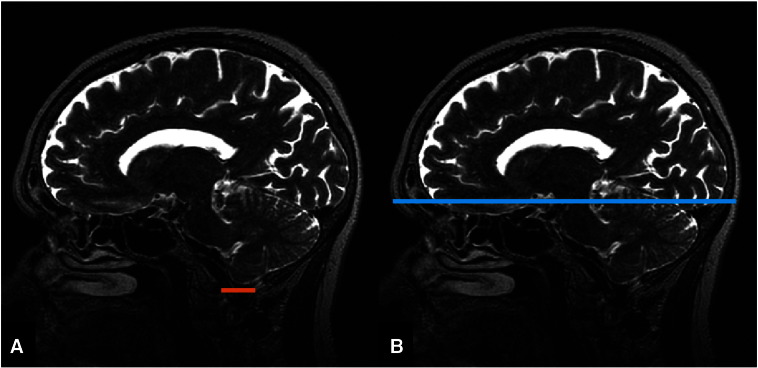
(A) sagittal slice from a T2W MRI sequence showing the inferior limit of the intracranial volume at the foramen magnum (orange). (B) identification of the inferior boundary using the upper edge of the supraorbital ridge (blue).

Measurements of current ICV were obtained using the T2*W sequence by a validated method [14]. The inferior limit of the intracranial cavity was defined as the axial slice that was superior to the tip of the odontoid peg at the foramen magnum (Fig. 1), and excluding the cavernous and extradural sinuses. To define the limits of the current ICV, we placed a seed-point in the axial slice at the midpoint where the orbits showed the optic nerve and selected the optimal threshold as the intensity value that separated the optic nerve from the rest of the brain tissue. The Object Extraction Tool (OET) in Analyze 9.0 that applies morphological erosion, dilation, region growing steps and thresholding, was used automatically to segment the ICV. The software then automatically extracted the ICV, creating a current ICV volume mask where the outer limit was the dural lining of the inner skull table. The current ICV mask was visually assessed and manually edited where necessary to exclude erroneous tissue using the MCMxxxVI multispectral segmentation tool [15] (http://sourceforge.net/projects/bric1936/). The MCMxxxVI method uses the colour combination of T2*W and FLAIR in the red/green colour spectrum, which facilitates easier identification of the brain boundaries in difficult areas. This method also determines cerebrospinal fluid (CSF) volume.

**Fig. 2:**
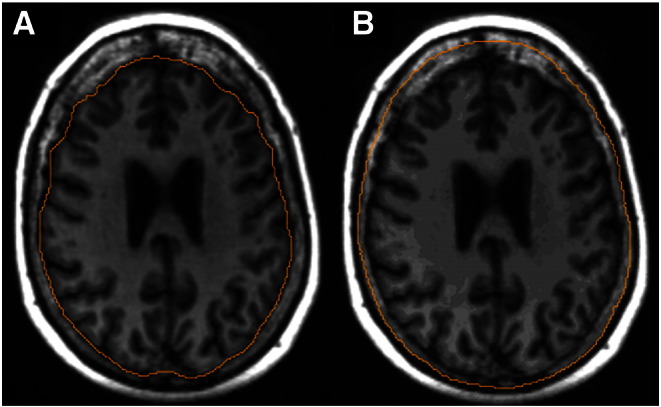
(A) ICV mask acquired using the MCMxxxVI method and providing the current ICV measurement. (B) ICV mask edited to provide the estimated original ICV excluding the effects of skull thickening.

We estimated the original ICV, denoted as ‘estimated original’ ICV, excluding the effects of inner table skull thickening by editing the current ICV mask slices throughout the skull vault, extending its boundaries to include the inner skull table thickening. The inferior boundary for the measurement of inner table skull thickening was identified as the supraorbital ridge, which is the most prominent point in the midline sagittal view [16]. This landmark was used as it is an easily identifiable point and separates the vault where most of the inner table thickening occurs from the frontal sinuses and orbits where there is little thickening and the boundaries which are also more difficult to measure. Using multiplanar display software (MRIcro; www.cabiatl.com/mricro/mricro/index.html; Fig. 1), the sagittal view was selected and the location of the supraorbital ridge was highlighted to indicate which axial slice was the most inferior limit of the region. Then, for all slices showing ICV and inner table skull thickening superior to the supraorbital ridge, the edge of the current ICV mask was extended by manually tracing along the line where the original skull table was thought to be, as shown in Fig. 2. The inferior slices remained the same as those in the current ICV mask. The entire mask was re-measured providing an estimate of original ICV measurement without the effects of inner table skull thickening.

Finally, the current brain volume was measured in all subjects and brain atrophy determined by calculating the total brain tissue volume as a percentage of both current and estimated original ICV.

### 2.4 Statistical analysis

The sample was not normally distributed (Shapiro-Wilk normality test) for either the current (W = 0.902, p = 0.001) or estimated original ICV (W = 0.919, p = 0.001). The Wilcoxon Signed Ranks test was used to identify differences between current and estimated original ICV across the whole group. The Mann–Whitney U test was applied for differences between men and women. To test the potential effect that thickening of the inner skull table would have on estimates of brain atrophy between youth and old age, the Wilcoxon Signed Ranks test was used to identify the differences between percent brain tissue in current and estimated original ICV in the whole group. The Mann–Whitney U was used to test for gender differences between percentage brain tissue in both measurements.

### 2.5 Inter- and intra-class correlations (ICC)

To assess within and between observer variability measurements of both the current ICV and estimated original ICV, repeat measurements of both were made by the same image analyst and by another analyst separately. For current ICV the intra-class correlation coefficient was 0.98 and the inter-class correlation coefficient was 0.96; and for estimated original ICV the intra-class correlation coefficient was 0.98 and the inter-class correlation coefficient was 0.98. This suggests the measurements have a good degree of reproducibility within and between observers.

## 3 Results

The median current ICV for the whole group was 1409.1 ml and the median estimated original ICV was 1480.1 ml. The median difference between the ICV measurements was 108.54 ml representing a percentage median difference of 7.3%, which was significant across the whole group (z = − 6.33; p < 0.001). Quartile ranges are presented in Table 1 and statistics for Wilcoxon Signed Ranks and Mann–Whitney U tests are shown in Table 2.

The median current and estimated original ICV in men (current ICV = 1643.5 ml and estimated original ICV = 1741.1 ml) were larger than in women (current ICV = 1228.5 ml and estimated original ICV = 1354.8 ml). The absolute difference between current and estimated original ICV was greater for women 114.6 ml (z = − 4.541; p < 0.001) than for men 101.9 ml (z = − 4.457; p < 0.001), confirming that women showed a greater decrease in ICV due to inner table skull thickening (8.3%) than did men (6.2%). However, when considering the difference between current and estimated ICV for men and women without adjusting for head size, the result was not significant (z = − 0.718; p > 0.05). Only when individual differences in head size were accounted for by correcting for current ICV prior to comparison, did the difference between current and estimated original ICV between men and women became significant (z = − 3.523; p < 0.001).

We assessed the percentage of total brain tissue in current (78.8%) and estimated original ICV (73.1%) as a measure of brain atrophy. The difference between the percentage of total brain tissue in current and estimated original ICV for the whole group (5.3%) was significant (z = − 6.334; p < 0.001). The percentage of total brain tissue in current ICV in men (76.7%) and women (80.9%) was significantly different (z = − 3.188; p < 0.001) making it appear that women had less brain atrophy than men. However, the percentage of total brain tissue in estimated original ICV between men (71.7%) and women (74.1%), was not significantly different (z = − 1.280; p > 0.05) indicating that there was no sex differences in the degree of brain atrophy once the effects of inner table skull thickening had been removed.

## 4 Discussion

Thickening of the inner skull table occurs with ageing and can significantly affect the measurement of ICV, and hence of estimated brain tissue loss in relation to the cranial cavity. The reduction in ICV is more pronounced in women, who on average have more inner table skull thickening than men, but the difference in head size between men and women artificially distorts the magnitude of this difference. The finding of significantly more inner table skull thickening in females (8.3%) than males (6.2%) is consistent with the literature on physiological changes of the skull with age [9]. The significant difference in current ICV measurements between men and women reflects the fact that men have larger heads than women, but the disappearance of the significant difference when the effect of skull thickness is removed highlights that the extent of skull thickening is greater in women than in men.

The influence that inner table skull thickening can have on estimating brain atrophy is significant and could be a potential problem for estimates of brain tissue loss in ageing. Our findings show that gender differences in atrophy are affected by inner table skull thickening and may be obscuring true differences in brain atrophy with age [18,19], or in estimates of original brain size in youth to compare with cognition in old age. This is an important consideration when attempting to approximate whether men or women suffer more age-related brain tissue atrophy. The importance of using ICV as a covariate has been aptly displayed in the study by Scahill et al. [17] which looked at brain volume changes in normal ageing, and found that significant gender effects were lost when ICV was used to correct for differences in head size. The mechanism behind inner table skull thickening may be related to brain tissue loss in old age and although its cause is unclear, it remains an unexplored source of variance in gender comparisons and ageing research.

The strengths of this study are that we used exemplar subjects chosen to represent a range of degrees of inner skull table thickening from a well characterised older cohort. We performed all analyses blind to all other subject information and used well-validated image processing tools and checked the outputs visually and corrected any erroneous tissue inclusion/exclusion manually. We made careful attempts at standardising the measurement and increasing the reproducibility by choosing an easily identifiable inferior boundary, aligning all images to the AC-PC line.

**Fig. 3:**
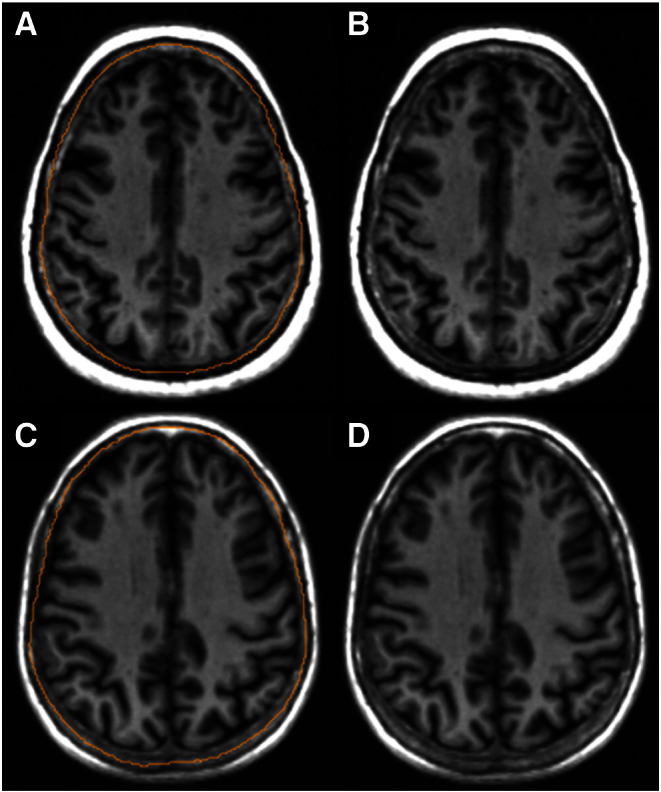
Example of the difficulty identifying the original inner skull table prior to the effects of skull thickening in those participants with some thickening (A, B), and those participants with little thickening (C, D).

The limitations include the relatively small sample, the subjective decision necessary to delineate the original inner skull table, which was difficult to identify consistently in people with any moderate inner table skull thickening (Fig. 3), and uncertainty about the generalisability to other populations in which the degree of inner table skull thickening may be even greater. Clearly, having identified the potential scale of the problem, it is now necessary to direct future research towards finding ways of estimating original ICV reliably.

Although the pathological implications of inner skull table thickening may not be of great medical research interest due to its presumed benign nature, the influence this thickening has on estimates of brain atrophy in studies of ageing and potentially in clinical practice should be considered. Inner table skull thickening, while not present in all older people, can have considerable influence on estimates of brain atrophy, especially in woman. These findings call attention to an otherwise overlooked aspect of research into brain ageing and further informs research concerning gender differences. Though individual differences in inner skull table thickness are difficult to extrapolate from this relatively small sample, the demonstration that large degrees of thickening are present in older people is important. Presentation of results showing that these differences could obscure gender differences in brain atrophy further strengthens the implications of our findings.

## Figures and Tables

**Table 1:** Median and interquartile ranges of ICV and brain atrophy measures for the whole group, men and women.

Measurement	Group	N	Median	25th percentile	75th percentile
Current ICV (ml)	Whole group	60	1409.10	1229.44	1648.04
	Male	31	1643.46	1480.38	1698.79
	Female	29	1228.54	1199.03	1265.87
Estimated original ICV (ml)	Whole group	60	1480.16	1355.49	1747.41
	Male	31	1741.12	1602.81	1820.38
	Female	29	1354.75	1290.5	1381.23
Absolute difference in ICV (ml)	Whole group	60	108.54	86.74	131.18
	Male	31	101.98	79.42	131.92
	Female	29	114.61	88.31	129.33
% Brain tissue in current ICV	Whole group	60	78.84%	75.04%	81.40%
	Male	31	76.67%	74.29%	79.50%
	Female	29	80.93%	78.05%	82.48%
% Brain tissue in estimated original ICV	Whole group	60	73.10%	70.63%	76.04%
	Male	31	71.71%	70.26%	75.02%
	Female	29	74.08%	71.41%	76.32%
Absolute difference between	Whole group	60	5.29%	4.41%	6.70%
% brain tissue in current and	Male	31	4.74%	3.48%	5.50%
estimated original ICV	Female	29	6.55%	5.07%	7.63%

**Table 2:** Test score (Z) and p-value for the difference (Mann–Whitney U and Wilcoxon signed Rank) between measurements within groups and within measurements between groups.

Difference between current and estimated original ICV (Wilcoxon signed rank test)	Whole group	Z = − 6.334; p < 0.001
	Males	Z = − 4.457; p < 0.001
	Females	Z = − 4.541; p < 0.001
Difference in current and estimated original ICV between males and females (Mann–Whitney U test)	-	Z = − 0.718; p > 0.05
Difference in current and estimated original ICV between males and females after correcting for head size using current ICV (Mann–Whitney U test)	-	Z = − 3.523; p < 0.001
Difference in % brain tissue in current and estimated original ICV (Wilcoxon signed rank test)	Whole group	Z = − 6.334; p < 0.001
Difference in % brain tissue in current ICV between males and females (Mann–Whitney U test)	-	Z = − 3.188; p < 0.001
Difference in % brain tissue in estimated original ICV between males and females (Mann–Whitney U test)	-	Z = − 1.280; p > 0.05
